# Leech Extract Enhances the Pro-Angiogenic Effects of Endothelial Cell-Derived Exosomes in a Mouse Model of Ischemic Stroke

**DOI:** 10.3390/cimb47070499

**Published:** 2025-07-01

**Authors:** Yushuang Cao, Jin Sun, Lichen Guo, Meng Wang, Linlin Su, Tong Zhang, Shaoxia Wang, Lijuan Chai, Qing Yuan, Limin Hu

**Affiliations:** State Key Laboratory of Component Chinese Medicine, Tianjin University of Traditional Chinese Medicine, Tianjin 071000, China; caoyushuang456@163.com (Y.C.); sunjin0924199@163.com (J.S.); chen18434376639@163.com (L.G.); wangmeng02022@163.com (M.W.); sututu0514@163.com (L.S.); tjutongnn@163.com (T.Z.); wangshaoxia1@163.com (S.W.); cljuan1258@126.com (L.C.)

**Keywords:** MCAO/R, endothelial exosome, leech extract, miRNA, collateral circulation, angiogenesis

## Abstract

Background: Intercellular communication, facilitated by exosomes (Exos) derived from endothelial cells (ECs), significantly influences the regulation of angiogenesis. Leech extract significantly reduces ischemia–reperfusion injury, promotes angiogenesis, and improves neurological function in mice with stroke. However, further investigation is required to determine whether leech promotes angiogenesis through EC-Exo. Objective: This study aims to further explore whether leech regulates Exos to promote the establishment of collateral circulation in mice with ischemic stroke (IS) and the specific mechanisms involved. Methods: Here, we utilized an in vitro co-culture system comprising ECs and pericytes to investigate the impact of Leech-EC-Exo on enhancing the proliferation and migration of mouse brain microvascular pericytes (MBVPs). We further established an in vivo mouse model of middle cerebral artery occlusion/reperfusion (MCAO/R) to investigate the effects and underlying mechanisms of leech on collateral circulation establishment. Results: The findings demonstrated that leech significantly enhanced the in vitro cell migration number and migration number of pericytes. Therefore, it can also enhance the effect of EC-Exo on improving the infarct area and gait of mice, as well as modulating the HIFα-VEGF-DLL4-Notch1 signaling pathway to promote cerebral angiogenesis and facilitating the stable maturation of neovascularization in vivo. Conclusions: These results suggest that leech has the potential to enhance collateral circulation establishment, and its mechanism may involve the modulation of miRNA content in Exos and the promotion of signaling pathways associated with angiogenesis and vascular maturation.

## 1. Introduction

Stroke is a prevalent condition affecting one in four individuals during their lifetime [1]. The majority of strokes are ischemic in nature, resulting from reduced blood flow primarily caused by arterial occlusion. At present, the management of ischemic stroke includes tissue plasminogen activator (tPA), mesenchymal stem cell (MSCs) therapy, nanozymes, the gut–brain axis in traditional Chinese medicine, and other approaches [2,3,4]. tPA is the sole FDA-approved medication for treating IS as it effectively dissolves blood clots [5]. This therapy is subject to several limitations, including a narrow treatment window of only 4.5 h, beyond which it may potentially result in cerebral hemorrhage [6]. Studies have demonstrated that effective collateral circulation can restrict the expansion of the ischemic core and extend the duration during which penumbral tissue at risk remains amenable to salvage until reperfusion therapy is administered [7,8,9,10]. Under hypoxia and other stimuli, the induction of neovascularization, as a tertiary type of cerebral collateral circulation system [11], can enhance cerebral blood supply through the generation of new blood vessels. Therefore, rejuvenating angiogenesis constitutes the principal strategy for reinstating collateral circulation and enhancing blood perfusion in ischemic tissue.

The process of angiogenesis is a multifaceted biological phenomenon, encompassing diverse cellular components, molecular mediators, and intricate signaling cascades [12,13,14]. The key cellular components involved in angiogenesis encompass ECs and pericytes, with their intercellular communication playing a pivotal role in the establishment, maintenance, and functionality of vascular networks [15,16]. Exos, a subtype of extracellular vesicles [17,18,19], possess the unique ability to traverse physiological barriers including the blood–brain barrier without eliciting tumorigenic effects, thereby serving as potent mediators for long range intercellular communication. It is specific that EC-Exo can maintain the integrity of the cerebrovascular barrier by transmitting biological signals to neighboring cells [20]. However, the underlying mechanism by which EC-Exo functions as a means of communication to stimulate pericytes and enhance angiogenesis remains elusive.

Promoting angiogenesis in the infarcted area, thereby restoring blood supply and reducing neurological deficit, aligns with the concept of smoothing the veins of the brain in TCM. Leech is a representative animal-based drug for promoting blood circulation and removing blood stasis [21]. It has definite therapeutic effects in anticoagulation, antithrombosis, and the improvement of brain nerve injury. It can improve endothelial cell injury in patients, inhibit the expression of inflammatory factors and the development of oxidative stress, and also reduce programmed cell death such as apoptosis. It achieves brain nerve protection from multiple dimensions and its long-term efficacy in the prevention and treatment of ischemic stroke is worthy of attention. According to Shennong’s herbal classics, dried whole *Whitmania pigra Whitman*, *Hirudo nipponica Whitman*, or *Whitmania acranulata Whitman* leeches are often used to treat abdominal masses, stroke hemiplegia, traumatic injury, and other syndromes. The active components derived from leech have been demonstrated to possess indispensable therapeutic potential in the treatment of IS by exerting effects such as anti-inflammatory and anticoagulant actions, the enhancement of microcirculation, the promotion of angiogenesis, and neuroprotection [22,23,24]. Hirudin exhibits the ability to enhance EC proliferation and vascular endothelial growth factor secretion, thereby facilitating angiogenesis [25]. Our preliminary research findings demonstrated that the administration of leech exhibits a significant reduction in cerebral infarction volume while concurrently facilitating neurological function recovery and promoting angiogenesis in MCAO/R mice. However, the potential of leech to enhance intercellular communication and angiogenesis through EC-Exo remains uncertain.

In the present study, we established an in vitro endothelial cell–pericyte co-culture system to model oxygen–glucose deprivation/reoxygenation (OGD/R), and an in vivo MCAO/R model in mice. We conducted investigations to determine whether leech can enhance the proliferation and migration of pericytes through EC-Exo, employing cell migration assays and cell scratch assays. Subsequently, we employed TTC staining, modified neurological severity score (mNSS), gait tests, and HE staining to assess the therapeutic efficacy of leech in ameliorating MCAO injury in mice. Additionally, immunofluorescence, laser speckle imaging, and an in vivo imaging system were utilized to investigate the angiogenic potential of Leech-EC-Exo. Relevant to the exploration of its mechanism, Western blotting (WB) and differential miRNA analysis were employed.

## 2. Materials and Methods

### 2.1. Extraction and Identification of EC-Exo

#### 2.1.1. Extraction of EC-Exo

EC-Exo was extracted and separated in accordance with our previous investigation [26]. The cell supernatant was subjected to repetitive centrifugation under conditions of 4 °C and 3000× *g* for 10 min, 2000× *g* for 20 min, and 10,000× *g* for 30 min in order to eliminate impurities (#XPN-100, BeckmanCoulter, Brea, CA, USA). Then, the sample was subjected to ultrafiltration and concentration using an ultrafiltration tube (#UFC900396, Millipore, Burlington, MA, USA) with a molecular weight cut-off (MWCO) of 100 kDa, and recentrifuged twice at 4 °C and 100,000× *g* for 70 min. Finally, it was resuspended with PBS solution and stored at −80 °C.

#### 2.1.2. Identification of EC-Exo

The morphology of EC-Exo was examined using a transmission electron microscope (#HT7800, Hitachi, Chiyoda, Japan) in this study. Prior to that, a 10 μL suspension of Exo was applied onto a copper mesh and allowed to stand for 2 min. Subsequently, an additional 10 μL of 2% phosphotungstic acid was introduced for another 2 min. After a further 10 min period of inactivity, the morphology could be observed using transmission electron microscopy (TEM). The particle size of EC-Exo was analyzed using a dynamic light-scattering particle (DLS) size analyzer (#MalvernNanoZS, Malvern, UK). Additionally, a BCA protein assay kit (#20150827, Thermo Fisher Scientific, Waltham, MA, USA) was employed for quantifying the EC-Exo marker protein after each extraction and before each experiment to ensure the homogeneity of exosomes among different batches and the comparability between groups.

### 2.2. In Vitro Experiments

#### 2.2.1. Cell Culture

The mouse brain endothelial cell line bEnd.3 (#CRL-2299, ATCC, Manassas, VA, USA) was procured for the study. The cells were cultured in freshly prepared Dulbecco’s Modified Eagle’s Medium (DMEM) (#C11995500BT, Gibco, Grand Island, NY, USA) supplemented with 10% FBS (#10099-141C, Gibco, Grand Island, NY, USA) and 1% PS (#15140-122, Gibco, Grand Island, NY, USA), and incubated at 37 °C in a CO_2_ cell incubator (#HERAcell150i, Thermo Fisher Scientific, Waltham, MA, USA). When the cell density was greater than 80%, we passaged the cells twice at a ratio of 1:4, adjusted the cell density to 1.2 × 10^5^ cells/mL, and inoculated them into 96-well plates. After 12 h of culture, Hoechst 33,258 (#C1011, Beyotime, Shanghai, China) was used for mycoplasma testing, and if qualified they could be used for subsequent experiments. Similarly, the MBVP strain was obtained from BNCC (#342014, BNCC, Beijing, China) and cultured following the same protocol.

#### 2.2.2. Cell Grouping and Drug Administration

When the cells were exposed to hypoxia, we rinsed them three times with PBS. We replaced the culture medium with standard DMEM in the Control group and glucose-free DMEM (#11966-025, Gibco, Grand Island, NY, USA) in the OGD/R group as well as the Leech groups. Upon the reoxygenation of the cells, the culture medium was replaced with fresh standard DMEM in the Control group and OGD/R group and varying dosages of leech extract in the Leech groups.

For bEnd.3: Based on our previous experiment, when cells were deprived of oxygen, the well plate was placed in a three-gas incubator (#MCO-5M, PHCbi, Chiyoda-ku, Japan) maintained at 37 °C with an atmosphere consisting of 2% O_2_, 5% CO_2_, and 93% N_2_. Oxygen deprivation was considered to occur when the O_2_ concentration dropped to 2%. After a four-hour period of hypoxia, the cells were taken out from the three-gas incubator and reoxygenated for 20 h in a standard cell incubator.

For MBVP: Similarly, the OGD/R model in the MBVPs was replicated using the same method as described above. The only distinction lay in the duration of oxygen and sugar deprivation, which lasted for 6 h, while reoxygenation was extended to 18 h.

#### 2.2.3. Cells Viability Assays

For the screening of leech dosage for bEnd.3 administration, leech extract was provided by Mudanjiang Youbo Pharmaceutical Co., Ltd. (Mudanjiang City, China). It was made by the same preparation process as Shuxuetong Injection with a concentration of 0.51 g/mL and confirmed to contain compounds such as hirudin, bufrudin, haemadin, and whitmanin. The experimental groups consisted of a Control group and Leech groups. Leech (#210608ST, Mudanjiang YouBo, Mudanjiang City, China) was diluted with DMEM to obtain concentrations of 2, 4, 8, 16, 32, 64, and 128 mg/mL. Upon reaching 80% coverage rate, the Control group was supplemented with DMEM, while the Leech groups were supplemented with leech, followed by the assessment of cell viability after a 24 h incubation period.

For the screening of EC-Exo dosage for MBVP administration, the MBVPs were modeled as previously described and subsequently treated with two types of EC-Exo at doses of 20, 40, 80, and 120 μg/mL during the reoxygenation phase [26]. Subsequently, cell viability was assessed.

Then, CCK-8 was employed for assessing cellular viability in numerous experiments. Following the manufacturer’s instructions, the culture medium was discarded and substituted with 10% CCK-8 (#40203ES60, Yisheng, Tianjin, China). The absorbance at 450 nm was measured using a microplate reader (#ZX21, MolecularDivices, San Jose, CA, USA) after incubation at 37 °C in the absence of light for a specified duration.

#### 2.2.4. Assessment of Uptake Capacity of MBVP Uptake to EC-Exo

Exos were labeled with the orange fluorescent film dye CM-Dil (#C7000, Thermo Fisher Scientific, Waltham, MA, USA) [27]. The prepared dye was mixed at a final concentration of 1 μM with EC-Exo at a concentration of 5000 μg/mL in a 1:1 ratio, followed by incubation at 37 °C for 30 min. The excess dye was subsequently removed using PBS. CM-Dil-labeled EC-Exo was then added to the medium for culturing MBVPs and incubated in darkness for 12 h. Finally, phalloidin (#YP0059S, Uelandy, Suzhou, China) was employed for cytoskeleton staining and Hochest33342 (#62249, Thermo Fisher Scientific, Waltham, MA, USA) was used for nuclear staining. The INCellAnalyzer2500HS system (#INCell 2500HS, Cytiva, Marlborough, MA, USA) was used for observation and cell imaging analysis.

#### 2.2.5. Transwell Experiments

According to former research [28], we established a co-culture system of ECs and pericytes. Transwell chambers were placed in a 6-well plate and 1 mL of MBVP suspension was evenly added to the upper layer, while DMEM culture medium was added to the lower layer to maintain consistent liquid levels between the internal and external compartments. Meanwhile, a 2 mL suspension of bEnd.3 cells was inoculated into another 6-cell plate. Approximately one day later, the MBVPs were subjected to oxygen–glucose deprivation, resulting in their impairment. During reoxygenation, the transwell chambers were transferred to the 6-well plate previously inoculated with bEnd.3 cells and supplemented with DMEM to ensure equal liquid levels between the inner and outer compartments. At same time, leech, leech + GW4869 (#6823-69-4, MCE, Dallas, NJ, USA), and GW4869, an exocrine inhibitor, were given to the lower layers, in their respective administration groups for further culture for 24 h.

The migration ability of MBVPs was assessed using crystal violet staining. Initially, the cells in the upper layer of the transwell chamber were carefully removed, followed by two washes with PBS. The cells were fixed using 4% paraformaldehyde (#P0099, Beyotime, Shanghai, China) and subsequently stained with 0.1% crystal violet stain (#C0121, Solarbio, Beijing, China) at room temperature for a duration of 20 min. Cell migration was observed using an inverted phase contrast microscope (#CKX53, Olympus, Tokyo, Japan), and the crystal violet staining solution was decolorized with 33% acetic acid (#64-19-7, Sinopharm Chemical Reagent, Beijing, China). The absorbance of the eluent at 570 nm was measured.

#### 2.2.6. Cell Scratch Assays

The co-culture system was established by the method mentioned above. Following OGD-induced injury to the MBVP cells, during reoxygenation, a vertical demarcation line was drawn in the center of the plate and subsequently washed with PBS to remove detached cells. Simultaneously, the transwell chambers were transferred to a 6-well plate pre-inoculated with MBVP cells, and the inner and outer liquid levels were equalized by adding DMEM. Subsequently, leech, leech + GW4869, and GW4869 were individually administered to the lower compartments of the experimental groups for a further 24 h of culture.

### 2.3. In Vivo Experiments

#### 2.3.1. Animals

Prior to the experiment, 56 male C57BL/6J mice weighing 23–25 g were procured from Beijing Viton Lever Laboratory Animal Technology Co, Ltd. (Beijing, China). They were housed under controlled conditions of a 12 h light–dark cycle at a temperature of 25 °C and provided with standard rodent chow and ad libitum access to drinking water. The animal experiments conducted in this study were performed in strict accordance with the relevant regulations set forth by the National Institute of Health and adhered to the Guidelines for the Care and Use of Experimental Animals. Furthermore, ethical approval was granted by the Animal Care and Use Committee of Tianjin University of Traditional Chinese Medicine (Permit No. TCM LAEC2021246, 14 November 2021, Tianjin, China).

#### 2.3.2. Establishment of Mouse MCAO/R Model and Leech Treatment

According to the previous research, the MCAO/R model in mice was reproduced by the thread embolism method [29]. Before the operation, the mice were fasted for 12 h and free to drink water. During the operation, isoflurane (#R510-22-10, RWD, Shenzhen, China) was used to ensure the full-time anesthesia of the mice, and an electrothermal thermostat (#ALC-HTP, Alcbio, Shanghai, China) was used to maintain the rectal temperature of the mice at 37 ± 0.5 °C. Then, the blood vessels and muscles of mice were dissected to expose the common carotid artery (CCA), internal carotid artery (ICA), and external carotid artery (ECA), followed by ligation at the proximal end of the CCA and distal end of the ECA. The thread plugs were carefully advanced into the middle cerebral artery (MCA) following the ECA-ICA direction, and their progression was halted upon encountering minimal resistance. This precise positioning ensured the optimal occlusion of MCA blood flow by the thread plug. The Sham group had all the operations without inserting thread plugs. After a 1.5 h period of ischemia, the thread plugs were carefully removed and the mice were subsequently raised under normal conditions upon complete awakening.

Intra-operative monitoring: During the entire surgical procedure, the vital signs of mice, such as breathing, heart rate, and body temperature, were closely monitored. If any abnormality is detected, the operation was immediately halted and appropriate measures were taken promptly.

Exclusion criteria: A total of 4 h after reperfusion, the neurobehavioral results of the mice were scored by Zea Longa score [30], which is scored on a five-point scale. Mice with scores of 1–3 were considered as success cases of MCAO, and those with 0 or 4 were excluded from this research.

The successful mice were randomly allocated into the Model group, EC-Exo group (60 μg/mouse) [26,31,32,33], and Leech-EC-Exo group (60 μg/mouse). Exosomes were administered 2 h after reperfusion, which corresponded to the timing of most stroke patients’ interventions (144 min) [34]. The mice were intravenously injected with the test substance every other day seven consecutive times, while the Control and Model groups received injections of PBS. The dosage and frequency of administration were determined based on previous research.

#### 2.3.3. Animal Euthanasia and Organ Collection

Following drug treatment, the mice were appropriately anesthetized and euthanized by the intraperitoneal injection of 1.2% tribromoethanol. According to various experiments, the methodologies for organ collection exhibit variations and are elaborated upon in each specific experimental approach.

#### 2.3.4. Modified Neurological Severity Score (mNSS)

The mNSS was employed to systematically assess motor nerve function impairment, encompassing aspects of nerve function, motor function, sensory perception, and balance ability on days 1, 3, 7, and 14 [35,36]. The neurological function rating ranged from 0 to 18, with mild injuries classified as scores 1–6, moderate injuries as scores 7–12, and serious injuries as scores 13–18. The specific scoring criteria are as follows:

(1) Motion test
  ① Tail suspension test:
    - 0 points for balancing;
    - 1 point for the flexion of a contralateral forelimb;
    - 2 points for the flexion of a contralateral hind limb;
    - 3 points for head turns away from the vertical axis by more than 10° within 30 s.
  ② Walking test:
    - 0 points for going straight normally;
    - 1 point for not being able to go straight normally;
    - 2 points for turning to the same side;
    - 3 points for falling on the same side as the paraplegia.
(2) Sensation test:
  ① Placement test—mouse placed on the edge of the table:
    - 0 points for forelimbs on the table;
    - 1 point for no forelimbs on the table.
  ② Proprioception test—mouse pushed to the edge of the table:
    - 0 points for resisting with the opposite forelimb;
    - 1 point for no resistance.
(3) Balance test—mouse placed on wooden strips with a width of 0.6 cm, height of 1.2 cm, and length of 120 cm:
    - 0 points for keeping static balance for greater than 60 s;
    - 1 point for grasping the edge of the balance beam tightly;
    - 2 points for holding the balance beam tightly and one limb sliding off the balance beam;
    - 3 points for holding the balance beam and two limbs sliding down or trying to keep balance for more than 60 s;
    - 4 points for holding the balance beam and two limbs sliding down or trying to keep balance for 40 ~ 59 s and then falling down;
    - 5 points for holding the balance beam and two limbs sliding down or trying to keep balance for 20 ~ 39 s and then falling down;
    - 6 points for falling off the balance beam in less than 20 s or not trying to keep balance.
(4) Reflection test
  ① Auricular reflex—touch the external auditory canal of mouse with a cotton swab:
    - 0 points for shaking head;
    - 1 point for not shaking.
  ② Corneal reflex—touch the cornea with cotton:
    - 0 points for blinking;
    - 1 point for not blinking.
  ③ Panic reflex—make a noise using fast-elastic cardboard:
    - 0 points for jumping;
    - 1 point for not jumping.
(5) Abnormal movement: 1 point for dystonia after epilepsy and myoclonia.

#### 2.3.5. Gait Analysis

The Digi gait system (#MSI-DIG-MS, MSI, Boca Raton, FL, USA) was used to capture and analyze mouse gait [37]. The mice underwent adaptability training three days prior to the experiment. During the formal assessment, red ink was applied to the soles of their feet, while black ink was used to paint their mouths. Subsequently, the gait of each mouse was recorded using the Digi gait imaging system. After ensuring the accuracy of the data, statistical analysis was conducted on various indices including swing duration, braking duration, propulsion duration, stride length, paw area, gait symmetry, and movement maladjustment coefficient using the MATLAB program (V9.12).

#### 2.3.6. Observation of Velocity of Erythrocytes in Microvessels

The flow velocity was observed using an in vivo imaging microscope (#IVIS^®^LuminaK, Perkinelmer, Waltham, MA, USA). Firstly, Rhodamine 6G (#252433, Sigma, St. Louis, MO, USA) was injected intravenously at a dose of 1 mg/mL into the mice. The mice were prepared for observation by opening a 3 mm × 3 mm cranial window located behind the anterior fontanel point. Subsequently, brain microvessels with diameters ranging from 30 to 50 μm and an approximately 200 μm length, exhibiting minimal bending, were visualized using a microscope. Flow velocity measurements were conducted utilizing Image-Pro Plus 6.0 software.

#### 2.3.7. TTC Staining

TTC staining was used to evaluate cerebral infarction in mice. A total of 24 h after MCAO/R, one mouse in each group was randomly selected, anesthetized with tribromoethanol, and euthanized. The brain was taken and cut into five 1 mm brain slices along the longitudinal direction. We then added 2% TTC (#T8877, Sigma, St. Louis, MO, USA) to stain the tissue and incubated it at 37 °C in the dark for 10min.

#### 2.3.8. Hematoxylin–Eosin (H&E) Staining

The histopathology of mouse brain tissue was examined by HE. The mice were anesthetized by intraperitoneal injections of 1.2% tribromoethanol. After fixation, the chest was opened, the perfusion needle was inserted into the apex of the heart, and the right atrial appendage was cut. The mice were perfused with precooled PBS buffer until the liver and limbs turned white, and then 20 mL of precooled 4% PFA solution was perfused. Their brains were taken out carefully, and paraffin sections (3 μm) were made 2 mm after the optic chiasm of the mouse brain tissue from 4 mice in each group. Then H&E staining was performed with an H&E kit (#C0105, Solarbio, Beijing, China) after sectioning. The sections were observed by an observer who was blinded to the experimental group allocation, and then 3 random areas were selected for imaging.

#### 2.3.9. Observation of Capillary Density

Capillaries were identified by FITC–dextran (50 mg/mL) [38,39]. The mice were injected with 0.2mL FITC–dextran (#60842-46-8, Sigma, St. Louis, MO, USA) intravenously on the 14th day after MCAO/R and euthanized 10 min later. Their brains were taken out carefully, cut into 20 μm coronal sections by a vibration microtome, and observed with a PannoramicMIDI slice scanning analyzer (#PannoramicMIDI, 3DHistech, Budapest, Hungary).

#### 2.3.10. Immunofluorescence Staining

BrdU/CD31 immunofluorescence staining was used to evaluate the neovascularization of mice from each group [40]. A total of 14 d after the operations, the brain tissues of the mice were taken out after PBS perfusion in the same way as in Section 2.3.8 and made into frozen sections with thicknesses of 10 μm and 20 μm. After acid treatment, alkali neutralization, antigen repair, and other pretreatments, the frozen sections were incubated with a primary antibody at 4 °C overnight and a secondary antibody avoiding light at room temperature for 1 h. Finally, the nuclei were stained with DAPI and angiogenesis was observed under a microscope.

#### 2.3.11. Western Blotting Analysis

The expression of target proteins was detected by WB. Mouse brain tissues were taken out after PBS perfusion in the same way as in Section 2.3.8, split with PMSF (#P8340, Solarbio, Beijing, China), and quantified by the BCA kit. By electrophoresis and electrical transfer we separated the proteins of the same quality from each group and transferred them to a PVDF membrane. After being sealed with 5% skimmed milk (#P0216, Beyotime, Shanghai, China) at room temperature for 1 h, these were incubated with a primary antibody at 4 °C overnight and a secondary antibody at room temperature for 2 h successively. The antibodies involved in this experiment were anti-HIFα (#ab179483, Abcam, Cambridge, MA, USA), anti-VEGFA (#ab32152, Abcam, Cambridge, MA, USA), anti-VEGFR2 (#26415-1-AP, Abcam, Cambridge, MA, USA), anti-DLL4 (#21584-1-AP, Abcam, Cambridge, MA, USA), and anti-Notch1 (#4380T, CST, Kansas, MA, USA). Finally, the proteins were observed in an imaging system (#AI600, GE, Evendale, OH, USA) after treatment with an ECL chemiluminescent solution (#1423002, Millipore, Burlington, MA, USA) and analyzed by ImageJ (V1.54k).

#### 2.3.12. Real-Time PCR

The expression of miRNA in EX-Exo was detected by RT-PCR. Total RNA was extracted and quantified by an ultramicro nucleic acid analyzer [41]. According to the instructions of the HiPure Exosome RNA Kit (#R4319-02, magen, Shanghai, China), the RNAs were reverse transcribed, amplified, and analyzed. The primers were purchased from Aikerui Biology Company (South Croydon, UK) and their sequences were mmu-miR-21a-5p (UAGCUUAUCAGACUGAUGUUGA), mmu-miR-214-3p (ACAGCAGGCACAGACAGGCAGU), mmu-miR-218-5p (UUGUGCUUGAUCUAACCAUGU), mmu-miR-34a-5p (UGGCAGUGUCUUAGCUGGUUGU), mmu-miR-15a-5p (UAGCAGCACAUAAUGGUUUGUG), mmu-miR-126a-5p (CAUUAUUACUUUUGGUACGCG), mmu-miR-210-3p (CUGUGCGUGUGACAGCGGCUGA), mmu-miR-486a-5p (UCCUGUACUGAGCUGCCCCGAG), and mmu-miR-375-3p (UUUGUUCGUUCGGCUCGCGUGA). The Ct values were obtained and ΔCt values, the differences between the Ct values of the target genes and internal reference genes, were calculated for data statistics.

### 2.4. Statistical Analysis

The statistical results were expressed as means ± SD. Prior to analysis, all the datasets were thoroughly examined to establish the necessary assumptions for variance model analysis. Statistical comparisons were carried out with SPSS 23.0 software. The data was analyzed by one-way ANOVA followed by multiple comparisons using Tukey’s post hoc test. In addition, non-parametric data were analyzed using Kruskal–Wallis analysis of variance, and Dunn’s test was used when statistically significant. *p* < 0.05 was considered statistically significant. Every possible comparison between the study groups was considered and applied in the results.

## 3. Results

### 3.1. Isolation and Identification of Exosomes

**Figure 1:**
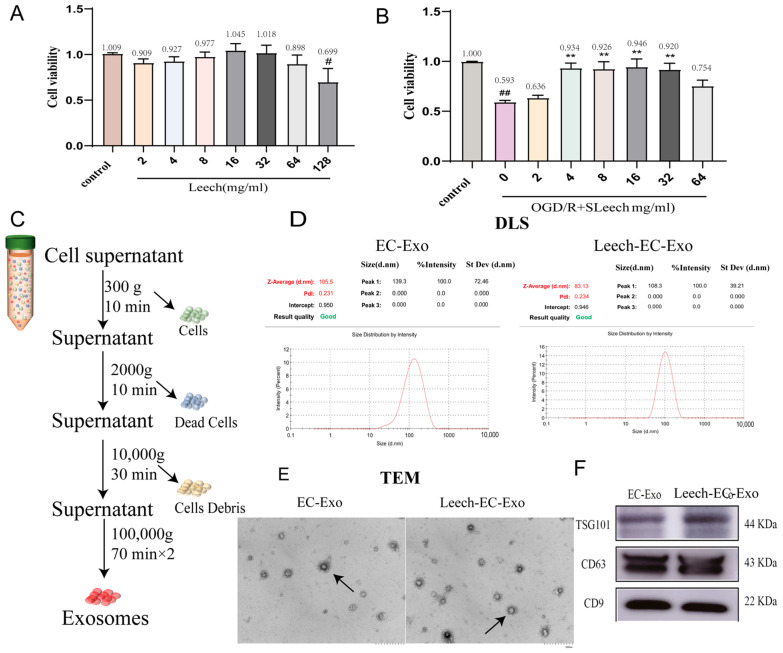
Isolation and identification of exosomes. (**A**) CCK-8 results of effect of leech on viability of normal cultured bEnd.3 cells (*n* = 5). (**B**) CCK-8 results of effect of leech on viability of OGD/R-injured bEnd.3 cells (*n* = 3). (**C**) Extraction and purification process of exosomes derived from brain microvascular endothelial cells (*n* = 3). (**D**) Detecting particle size distribution of Exos by DLS (dynamic light-scattering particle) (*n* = 3). (**E**) Identifying Exos by TEM (transmission electron microscopy). Scale bar = 500 nm (*n* = 3). The arrows represent the observed exosomes. (**F**) Identifying Exo marker proteins TSG101, CD63, and CD9 by WB (*n* = 3). Data analyzed by one-way ANOVA followed by multiple comparisons using Tukey’s post hoc test and expressed as mean ± standard deviation. # *p* < 0.05, ## *p* < 0.01 vs. control; ** *p* < 0.01 vs. OGD/R.

Firstly, a CCK-8 assay was performed to determine the optimal concentration of leech for normal and OGD/R bEnd.3 cells. The results indicated that leech had no significant effect on the viability of normal cultured bEnd.3 cells in the range of 2~64 mg/mL (Figure 1A), but 4~32 mg/mL could significantly improve the OGD/R viability of injured cells (*p* < 0.05) (Figure 1B). The concentration of leech for the follow-up experiments was set at 4 mg/mL. The Exos obtained through differential centrifugation exhibited consistent and high homogeneity (Figure 1C), as confirmed by TEM, DLS, and WB. The DLS results revealed that the average grain diameter of EC-Exo was measured to be 139.3 nm, while Leech-EC-Exo exhibited a slightly smaller average grain diameter of 108.3 nm (Figure 1D). EC-Exo and Leech-EC-Exo were observed via TEM as spherical structures with diameters ranging from 30 to 150 nm, which was consistent with the findings from DLS. No discernible differences in shape or size were observed between them (Figure 1E). The WB results revealed the expression of exosomal marker proteins TSG101, CD9, and CD63 in both EC-Exo and Leech-EC-Exo (Figure 1F).

### 3.2. Leech Enhances the Efficacy of EC-Exo in Facilitating the Proliferation and Migration of Pericytes

**Figure 2:**
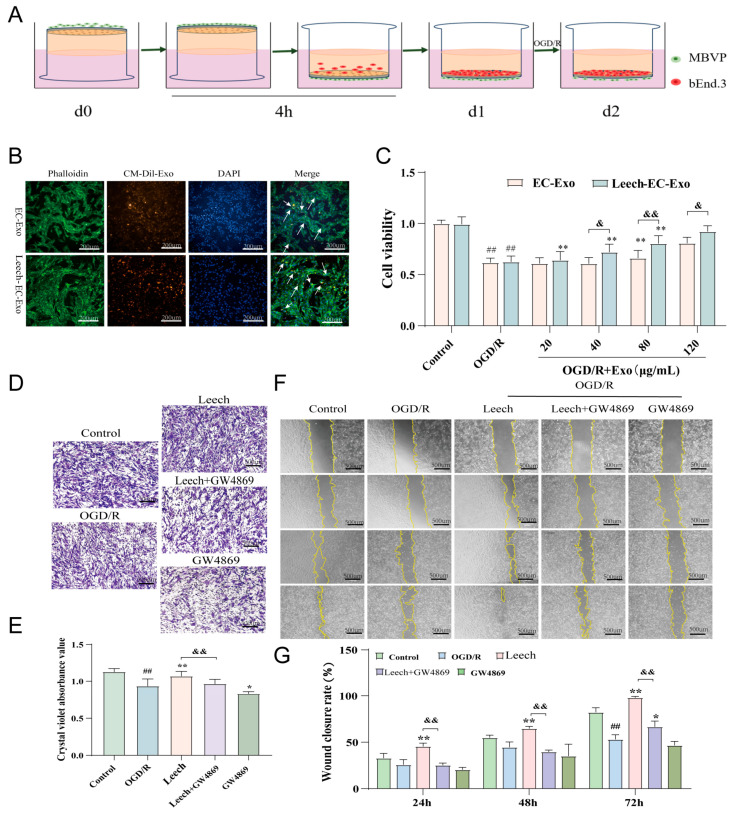
Leech enhances the efficacy of EC-Exo in facilitating the proliferation and migration of pericytes. (**A**) The establishment of the cell co-culture system. (**B**) The uptake of CM-Dil-labeled Exos by MBVPs (*n* = 3). The arrows represent the uptake of exosomes by pericytes. (**C**) The CCK-8 results of the effect of Exos on the viability of OGD/R MBVPs (*n* = 3). (**D**) Representative images of the transwell experiments (scale = 500 μm, *n* = 3). (**E**) A quantitative analysis of migration quantity (*n* = 3). (**F**) Representative images of the cell scratch assay (scale = 500 μm, *n* = 3). (**G**) A quantitative analysis of migration area (*n* = 3). The data was analyzed by one-way ANOVA followed by multiple comparisons using Tukey’s post hoc test and expressed as mean ± standard deviation. ## *p* < 0.01 vs. control; * *p* < 0.05, ** *p* < 0.01 vs. OGD/ROGD/R; & *p* < 0.0, && *p* < 0.01 vs. leech.

Firstly, the co-culture system of endothelial cells and pericytes was successfully established (Figure 2A). The ability of MBVPs to internalize EC-Exo and Leech-EC-Exo was assessed using immunofluorescence techniques. The results demonstrated that both EC-Exo and Leech-EC-Exo could be taken up by MBVPs (Figure 2B). The CCK-8 results revealed that Leech-EC-Exo significantly enhanced the activity of MBVPs following OGD/R injury at concentrations of 40, 80, and 120 μg/mL (*p* < 0.05) (Figure 2C). The migratory capacity of MBVP cells was assessed using transwell and scratch assays. The transwell results demonstrated a significant increase in the quantity of MBVP migration after leech treatment (*p* < 0.05). However, the addition of Exo inhibitor GW4869 resulted in a notable decrease in cell migration number (*p* < 0.05) (Figure 2D,E). The scratch results showed that the migration area increased significantly after leech treatment (*p* < 0.05) but decreased when GW4869 was added (*p* < 0.05) (Figure 2F,G). These findings suggest that leech may modulate pericyte proliferation and migration by regulating the secretion of EC-Exo, thereby implying its potential role in these processes.

### 3.3. Leech Enhances the Efficacy of EC-Exo in Improving Pathology in MCAO/R Mice

**Figure 3:**
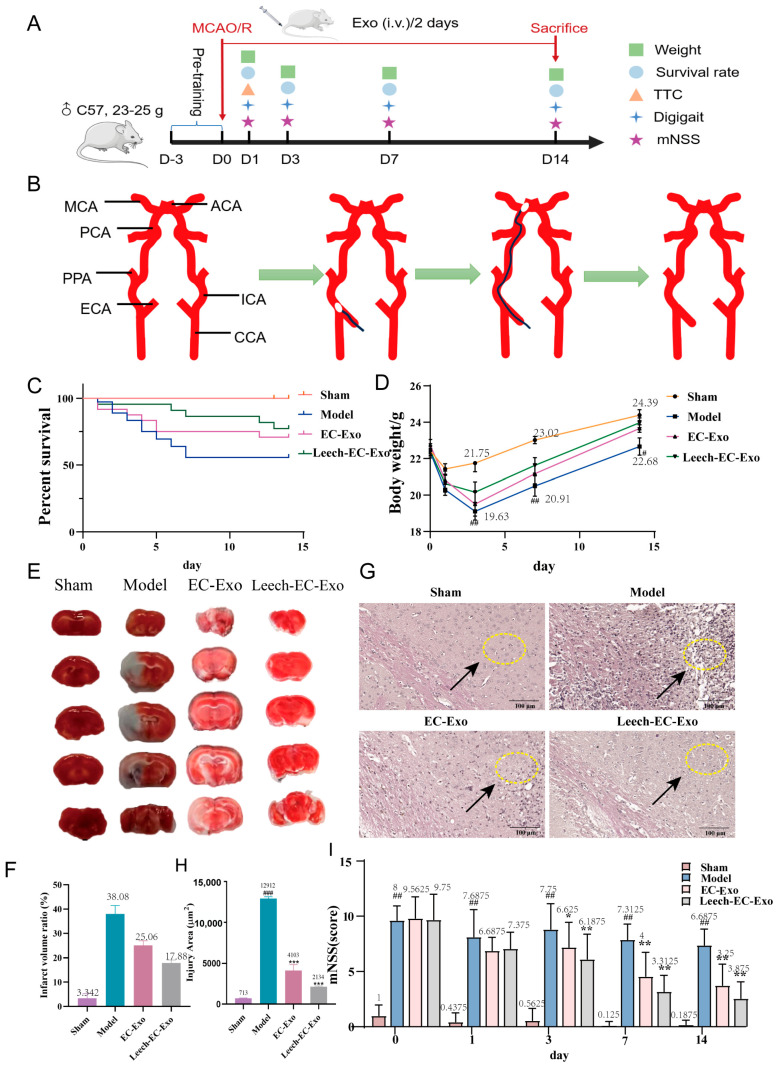
Leech enhances efficacy of EC-Exo in improving pathology in MCAO/R mice. (**A**) Design of animal experiments. (**B**) Establishment of MCAO/R model. (**C**) Survival rate within 14 days after MCAO/R (*n* = 14). (**D**) Weight changes within 14 days after MCAO/R (*n* = 14). (**E**,**F**) TTC stain examination of brain tissue 14 days after MCAO/R (*n* = 4). (**G**,**H**) H&E staining of pathological morphology of brain tissue 14 days after MCAO/R (*n* = 4). Both arrows and circles represent the situation of brain tissue damage. (**I**) Results of mNSS (*n* = 14). Data analyzed by one-way ANOVA followed by multiple comparisons using Tukey’s post hoc test and expressed as mean ± standard deviation. # *p* < 0.05, ## *p* < 0.01, ### *p* < 0.001 vs. Sham; * *p* < 0.05, ** *p* < 0.01, *** *p* < 0.001 vs. Model.

The animal experiments were designed in advance (Figure 3A). The establishment of the MCAO/R model is shown in Figure 3B. The weight and survival rate of the mice were recorded on the 1st, 3rd, 7th, and 14th day following the establishment of the MCAO/R model to assess the impact of Exos on mouse survival status. On day 14 post-operation, no mortality was recorded in the sham operation group, while varying degrees of mortality were observed among other mouse groups after MCAO/R modeling, primarily within 7 days post-operation. Particularly notable was a significant improvement in survival rates following exosome treatment, with a higher survival rate observed in the Leech-EC-Exo group compared to the EC-Exo group (Figure 3C). The results revealed a continuous decrease in mouse weight for three days post-operation in the Model group, followed by a gradual increase on the fourth day, which significantly differed from that observed in the Sham group (*p* < 0.01). During the 4–6th days after modeling, mice in the Leech-EC-Exo group exhibited weight gain following exosome treatment, with the lowest weight observed in this group being higher than that in both the Model and EC-EXO groups (Figure 3D). The TTC staining results further demonstrated that Leech-EC-Exo exhibited a significant reduction in the volume of cerebral infarction in MCAO/R mice, surpassing the efficacy of EC-Exo (Figure 3E,F). The brain tissue of MCAO/R mice exhibited prominent infarction in the HE staining images, characterized by disrupted cellular arrangement, cellular swelling or atrophy, the disappearance of nucleoli, the vacuolation of cells, and interstitial edema. After treatment with Leech-EC-Exo, the cells exhibited a more organized arrangement, accompanied by enhanced nucleoplasmic homogeneity, surpassing that observed with EC-Exo (Figure 3G,H). The impact of Leech-EC-Exo treatment on the motor function of MCAO/R mice was assessed using the mNSS neurological function score. The motor function, balance ability, sensory function, and reflex function of the mice exhibited varying degrees of abnormalities following the establishment of the MCAO/R model. Notably, from the third day after the administration of Exos, the Leech-EC-Exo group demonstrated a significant reduction in mNSS score (*p* < 0.05) (Figure 3I). The aforementioned findings collectively validate the neuroprotective efficacy of Leech-EC-Exo, thereby substantiating the potential of leech in regulating EC-Exo to facilitate post-stroke recovery in mice, enhance physical performance, and augment overall survival rates.

### 3.4. Leech Enhances the Efficacy of EC-Exo in Improving Motor Function in MCAO/R Mice

**Figure 4:**
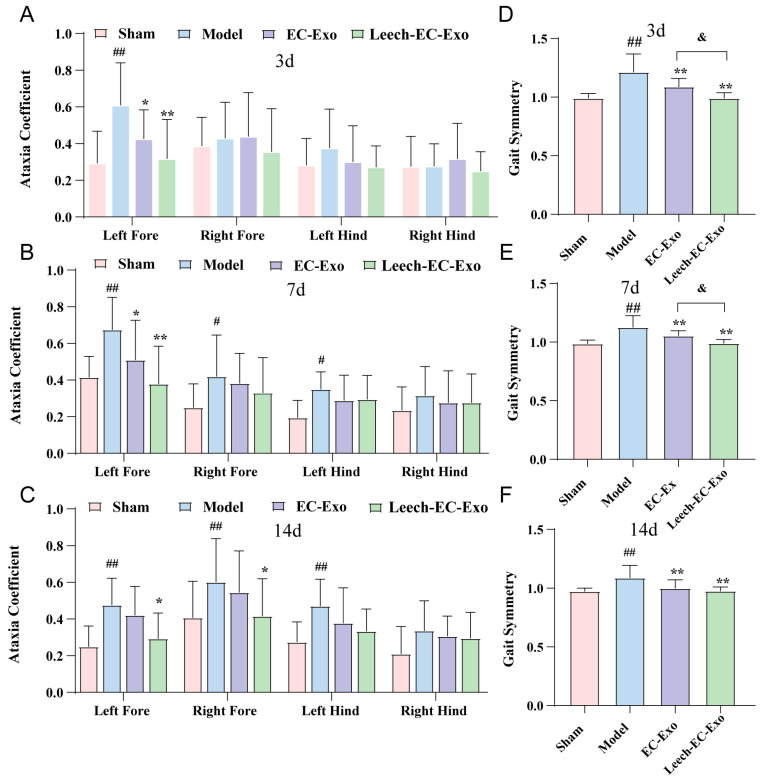
Leech enhances efficacy of EC-Exo in improving motor function in MCAO/R mice. (**A**) Analysis of ataxia coefficient of mice 3 days after MCAO/R (*n* = 14). (**B**) Analysis of ataxia coefficient of mice 7 days after MCAO/R (*n* = 14). (**C**) Analysis of ataxia coefficient of mice 14 days after MCAO/R (*n* = 14). (**D**) Analysis of gait symmetry of mice 3 days after MCAO/R (*n* = 14). (**E**) Analysis of gait symmetry of mice 7 days after MCAO/R (*n* = 14). (**F**) Analysis of gait symmetry of mice 14 days after MCAO/R (*n* = 14). Data analyzed by one-way ANOVA followed by multiple comparisons using Tukey’s post hoc test and expressed as mean ± standard deviation. # *p* < 0.05, ## *p* < 0.01 vs. Sham; * *p* < 0.05, ** *p* < 0.01 vs. Model; & *p* < 0.05 vs. EC-Exo.

Gait disturbance represents the primary motor impairment in stroke patients, frequently resulting in instability due to limb spasticity, muscle weakness, asymmetrical walking patterns, and reduced mobility, thereby heightening the risk of falls. To further validate the efficacy of Leech-EC-Exo in improving motor function in MCAO/R mice, we employed a gait analysis system to assess the ataxia coefficient and gait symmetry. The gait analysis revealed a significant increase in the ataxia coefficient of the MCAO/R mice, particularly in the left forelimb (*p* < 0.01). Treatment with Leech-EC-Exo demonstrated varying degrees of reduction in limb gait imbalance coefficients, notably in the left forelimb (*p* < 0.01). Notably, Leech-EC-Exo exhibited superior efficacy compared to EC-Exo (Figure 4A–C). Moreover, compared to the Sham group, the MCAO/R group exhibited deteriorated gait symmetry (*p* < 0.01). Following exosome treatment, the gait symmetry of the mice improved significantly, with Leech-EC-Exo demonstrating superior efficacy over EC-Exo (*p* < 0.05) (Figure 4D–F). These suggested that leech may enhance the efficacy of EC-Exo in ameliorating motor function in MCAO/R mice.

### 3.5. Leech Enhances the Efficacy of EC-Exo in Promoting Collateral Circulation Establishment in MCAO/R Mice

**Figure 5:**
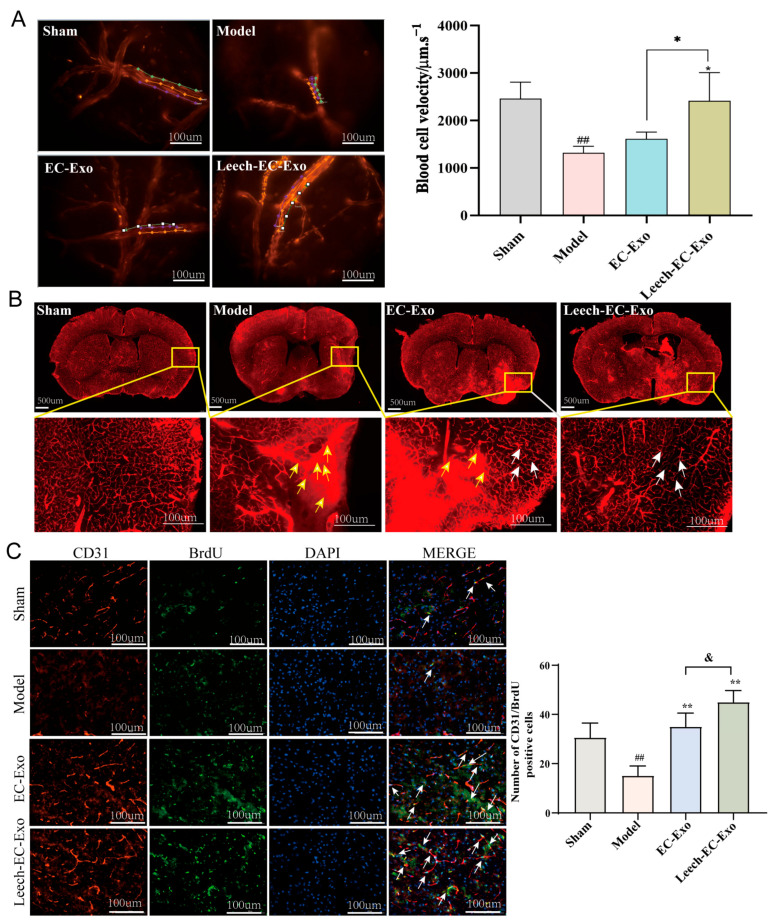
Leech enhances efficacy of EC-Exo in promoting collateral circulation establishment in MCAO/R mice. (**A**) Representative images depicting microvascular red blood cell velocity in MCAO/R mice along with corresponding statistical findings (*n* = 4). The lines represent the flow pathways of red blood cells. (**B**) Microvascular density and patency of MCAO/R mice (*n* = 4). The yellow arrows represent the accumulation of dyes, and the white arrows represent the improved distribution of blood vessels. (**C**) Representative images depicting immunofluorescence staining and corresponding statistical analysis of CD31/BrdU positive cells in ischemic cortex of MCAO/R mice (*n* = 4). Data analyzed by one-way ANOVA followed by multiple comparisons using Tukey’s post hoc test and expressed as mean ± standard deviation. ## *p* < 0.01 vs. Sham; * *p* < 0.05, ** *p* < 0.01 vs. Model; & *p* < 0.05 vs. EC-Exo.

The measurement of blood flow velocity in microvessels stands as a pivotal parameter within the realm of microcirculation research [42]. The results demonstrated that Leech-EC-Exo significantly enhanced the velocity of microvascular red blood cells in mice (*p* < 0.05), surpassing the efficacy of EC-Exo (*p* < 0.05) (Figure 5A). The brain microvascular density images revealed a significant reduction in vascular density within the infarcted areas and penumbras of the MCAO/R mice, accompanied by pronounced dye accumulation. Moreover, a notable disparity was observed between the left and right cerebral hemispheres. After Leech-EC-Exo intervention, the distribution of brain microvasculature exhibited greater homogeneity with reduced interhemispheric disparity. Moreover, compared to EC-Exo treatment, Leech-EC-Exo demonstrated superior efficacy (Figure 5B). The immunofluorescence results of CD31/BrdU indicated a more significant increase in the amount of neovascularization following Leech-EC-Exo intervention compared to EC-Exo treatment (*p* < 0.05) (Figure 5C). These findings suggest that leech can enhance the efficacy of EC-Exo in promoting the recovery of cerebral blood flow, improving blood flow dynamics, and facilitating the establishment of tertiary collateral circulation in MCAO/R mice.

### 3.6. Leech Modulates the Impact of EC-Exo on the HIFα-VEGF-DLL4-Notch1 Signaling Pathway in MCAO/R Mice

**Figure 6:**
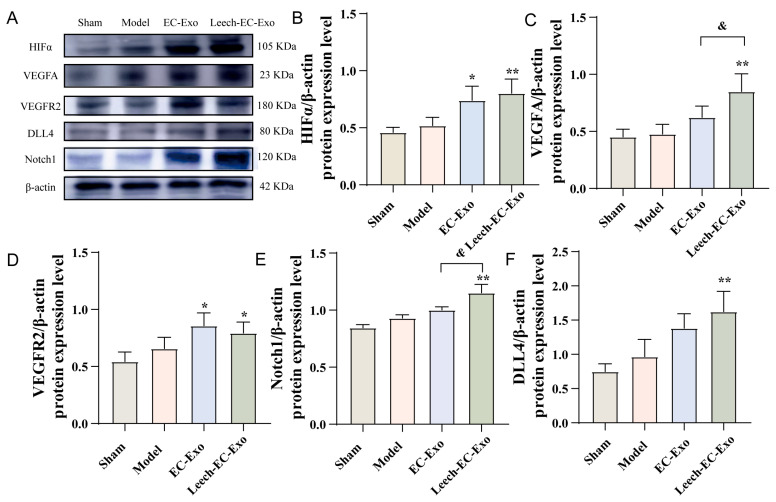
Leech modulates impact of EC-Exo on HIFα-VEGF-DLL4-Notch1 signaling pathway in MCAO/R mice. (**A**) Western blot detection bands of HIFα-VEGF-DLL4-Notch1 signaling pathway in MCAO/R mice (*n* = 4). (**B**) Quantitative analysis of HIFα protein in MCAO/R mice (*n* = 4). **(C)** Quantitative analysis of VEGFA protein in MCAO/R mice (*n* = 4). (**D**) Quantitative analysis of VEGFR2 protein in MCAO/R mice (*n* = 4). (**E**) Quantitative analysis of Notch1 protein in MCAO/R mice (*n* = 4). (**F**) Quantitative analysis of DLL4 protein in MCAO/R mice (*n* = 4). Data analyzed by one-way ANOVA followed by multiple comparisons using Tukey’s post hoc test and expressed as mean ± standard deviation. * *p* < 0.05, ** *p* < 0.01 vs. Model; & *p* <0.05 vs. EC-Exo.

Subsequently, we conducted WB to investigate the signal pathways associated with angiogenesis. The expression levels of HIFα, VEGFA, VEGFR2, DLL4, and Notch1 were significantly upregulated following Leech-EC-Exo treatment (*p* < 0.05), exhibiting a superior effect compared to EC-Exo in comparison to the Model group (*p* < 0.05) (Figure 6). The findings of this study suggest that leech can enhance the efficacy of EC-Exo in upregulating DLL4 protein expression, modulating VEGFR2 activity, and promoting angiogenesis and neovascularization stability in the ischemic cortex. The potential mechanism of this involves the activation of the HIFα-VEGF-DLL4-Notch1 signaling pathway through the upregulation of HIFα expression.

### 3.7. Leech Regulates the Species and Quantity of Angiogenesis-Related miRNA in EC-Exo

**Figure 7:**
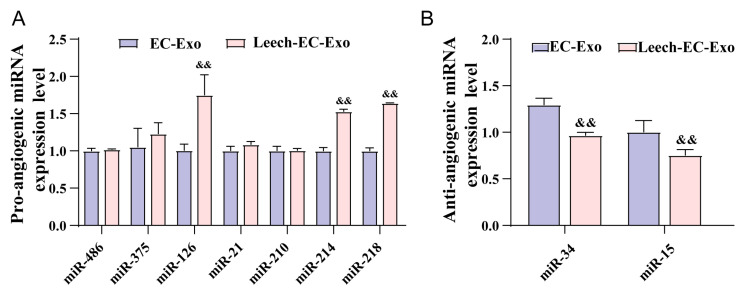
Leech regulates species and quantity of angiogenesis-related miRNA in EC-Exo. (**A**) Analysis results of pro-angiogenic microRNA expression level in EC-Exo and Leech-EC-Exo (*n* = 3). (**B**) Analysis results of anti-angiogenic microRNA expression level in EC-Exo and Leech-EC-Exo (*n* = 3). Data analyzed by one-way ANOVA followed by multiple comparisons using Tukey’s post hoc test and expressed as mean ± standard deviation. && *p* < 0.01 vs. EC-Exo.

To further elucidate the underlying mechanisms by which leech regulates angiogenesis through EC-Exo, an extensive review of the literature on angiogenesis was conducted, leading to the identification of nine specific miRNAs for subsequent experimental investigation (Table 1). The EC-Exo and Leech-EC-Exo samples were separately collected, followed by undergoing distinction analysis using RT-qPCR. Following stimulation with leech extract, there was a significant decrease (*p* < 0.05) (Figure 7A) in the levels of miR-15 and miR-296, which typically suppress vascular endothelial growth factor (VEGF) signaling and other pro-angiogenic pathways, thereby restricting blood vessel formation. The downregulation of miR-15 and miR-296 suggests that leech extract may alleviate their inhibitory effects, potentially facilitating angiogenic processes. Concurrently, the study revealed a notable upregulation (*p* < 0.05) (Figure 7B) in the expression of miR-126, miR-214, and miR-218, all of which are associated with promoting angiogenesis. miR-126, for instance, enhances VEGF and fibroblast growth factor (FGF) signaling, while miR-214 and miR-218 have been shown to stabilize endothelial cell function and stimulate vascular network formation. The increased abundance of these pro-angiogenic miRNAs further supports the hypothesis that leech extract actively shifts the miRNA profile of EC-Exo toward a pro-angiogenic state.

## 4. Discussion

Previous studies have demonstrated that leech, as a representative of animal medicine for promoting blood circulation to dispel blood stasis, has remarkable therapeutic efficacy in the treatment of IS. And EC-Exo can modulate nerve regeneration in MCAO/R mice and facilitate recovery from IS [26]. To further explore the effects and mechanisms of leech in the regulation of angiogenesis and its promotion of IS recovery through EC-Exo, the present study utilized in vitro and in vivo stroke models to demonstrate that leech possesses the ability to enhance angiogenesis through EC-Exo, thereby exerting neuroprotective effects. Further results suggested that this effect was highly correlated with the proliferation and migration of pericytes. A pivotal mechanism by which leech promotes angiogenesis is through the activation of the HIFα-VEGF-DLL4-Notch1 signaling pathway and modulation of miRNA content associated with EC-Exo.

Pericytes are situated on the inner side of the vascular basement membrane, intimately enveloping the capillary and microvessel endothelium, thereby exerting pivotal regulatory functions in angiogenesis and promoting its stabilization [60]. The ischemic penumbra tissue releases angiogenic factors following stroke to stimulate ECs in producing matrix metalloproteinases (MMPs) and degrading the basement membrane. Consequently, pericytes undergo activation and secrete cytokines such as PDGFRβ, TGF-β, and VEGF, thereby facilitating endothelial cell proliferation and promoting angiogenesis. An increasing number of experiments have substantiated the regulatory role of pericytes in EC barrier function and their ability to enhance connexin expression among ECs. The absence of pericytes leads to the disruption of the blood–brain barrier and a significant increase in vascular permeability [61,62,63,64,65]. However, we found that the migration ability of MBVPs was enhanced in the co-culture system with leech after OGD/R. The addition of GW4869 to inhibit EC-Exo secretion resulted in the inhibition of this effect, suggesting a crucial role for Exo in mediating cell communication between ECs and pericytes under leech mediation.

Several clinical studies focusing on ischemic stroke have highlighted that the establishment of collateral circulation can effectively decelerate the progression of penumbra tissue to infarction, thereby extending the treatment time window and mitigating the risk of hemorrhagic transformation [66,67,68,69]. Of note, our present study demonstrates that leech can facilitate the initiation of this process. BrdU, a crucial marker utilized to identify cells exhibiting proliferative activity during angiogenesis [40], is frequently co-labeled with specific markers of ECs and blood vessels to characterize regenerated vascular structures. The fluorescence intensity of CD31/BrdU in the ischemic cortex of MCAO/R mice was observed to significantly increase following Leech-EC-Exo treatment compared to EC-Exo, indicating its potential to enhance angiogenesis by modulating EC-Exo. In fact, our previous study demonstrated that leech can effectively enhance regional cerebral blood flow [26]. Moreover, leech administration significantly enhances collateral circulation establishment as evidenced by increased microvascular red blood cell velocity in MCAO/R mice. This finding is consistent with previous research demonstrating that a low concentration of hirudin enhances endothelial cell proliferation and vascular endothelial growth factor secretion, thereby promoting physiological angiogenesis. Furthermore, it facilitates neovascularization by improving the proliferation and migration of vascular endothelial cells, leading to an increased number of blood vessels and the formation of a new capillary network [25].

As a pivotal regulator, VEGF not only facilitates the process of angiogenesis [70], but also enhances the permeability of nascent blood vessels. The lack of endothelial gap junctions around the infarction area has been identified as a factor contributing to high permeability, which in turn promotes neovascularization and enhances the prognosis of stroke patients [71]. During the process of angiogenesis, VEGF plays a crucial role in the initiation and proliferation of tip cells through activation of the Notch signaling pathway [72]. As a crucial downstream gene of VEGF, DLL4 exerts regulatory control over the maturation and stability of neovascularization through the negative feedback modulation of VEGF. Our in vivo findings demonstrate that leech effectively activates the HIFα-VEGF-DLL4-Notch1 signaling pathway in MCAO/R mice, thereby enhancing angiogenesis and promoting the establishment of collateral circulation in the ischemic lateral cortex. This is achieved through the upregulation of key molecules including HIFα, VEGF, EGFR2, DLL4, and Notch1. In addition, Sen Zhang et al. [73,74] also discovered the pro-angiogenesis activity via the HIFα-VEGF-DLL4-Notch1 pathway. The development of drugs targeting VEGF seems to be an important direction for promoting the establishment of collateral circulation.

According to reports, Exos predominantly comprise miRNAs, which are considered as key constituents [75]. We profiled the miRNAs of Exos derived from ECs to further explore the molecular basis of the Exo effect we observed in this study. We identified nine miRNAs associated with ECs through an extensive review of the angiogenesis-related literature, and subsequently validated their expression in EC-Exo and Leech-EC-Exo using RT-qPCR. The results demonstrate that Leech-EC-Exo can upregulate the level of pro-angiogenesis-related miRNAs miR-126, miR-214, and miR-218, and downregulate the level of anti-angiogenesis-related miRNAs miR-15 and miR-296. This may mean that leech can promote angiogenesis via modulating the composition and abundance of miRNAs in EC-Exo. But we only verified this in Exo samples, not in tissue samples. Further studies could focus on the mechanisms of leech in promoting angiogenesis via regulating the miRNAs of Exos.

Overall, this study, in combination with the significant role of exosomes in angiogenesis, focuses on discussing how leech enhances the angiogenic capacity of exosomes, providing new ideas for the treatment strategies of stroke. In recent years, many studies have explored the role of exosomes in angiogenesis, but most of them have focused on stem cell-derived exosomes (such as MSC-Exo). However, issues like insufficient clinical indications for exosome treatment and limited sources for parental stem cells remain to be addressed [76]. In contrast, this study proposed a “leech extract regulation EC-Exo” strategy, whose advantage lies in natural medicine collaboration (leeches as traditional drug resistance; its extract may be modulated by many target body function secretions; avoid the limitations of single gene editing) and potential clinical transformation (EC-Exo derived from endothelial cells; combination with leech’s known security; more likely to promote clinical application), which may provide new ideas for treatment strategies for stroke.

Nevertheless, this study has potential limitations. For example, we have not explored the interaction between other brain cells and the underlying mechanisms of differential miRNAs, which could serve as crucial directions for future investigations. In addition, further investigation is warranted to unravel the precise role and functional mode of miRNAs within Exos. Furthermore, the active components in leech extracts (such as miRNA or proteins) may interfere with non-target pathways such as coagulation function or coagulation function after being delivered through exosomes. Further research can screen off-target molecules through methods such as single-cell RNA sequencing and verify their safety in animal models. It is worth noting that although we detected the content of exosomes between different batches through the BCA quantitative method in our experiments, in order to further ensure the stability of exosomes, the stability detection of miRNA and other aspects should also be involved. Our experiment only focused on the drug efficacy within 14 days after stroke, but the fibrotic risk of the drug and other factors still require longer-term observation.

## 5. Conclusions

In summary, our fundings demonstrated that leech effectively potentiated the impact of EC-Exo on the in vitro proliferation and migration of peripheral cells, while also promoting angiogenesis and augmenting the stable maturation of new blood vessels in the ischemic cortex in vivo. Its underlying mechanisms involve regulating the HIFα-VEGF-DLL4-Notch1 signaling pathway to facilitate angiogenesis and enhance the formation of functional collateral vessels, and miRNA content in Exos may also be involved. These novel findings on leech and Leech-EC-Exo could potentially serve as groundbreaking contributions to the fundamental research of traditional animal-based Chinese medicine, providing a solid foundation for its clinical application and facilitating new drug development.

## Figures and Tables

**Table 1:** miRNAs associated with angiogenesis.

Type of miRNA	miRNA	Target	Function	References
Pro-angiogenic	miR-126	VEGF	Stimulate VEGF-dependent AKT and ERK signal transduction to inhibit P85	[43]
		PI3K	Inhibit endothelial cell damage and promote angiogenesis	[44]
		Pcdh7	Promote cell proliferation and angiogenesis	
	miR-210	Ephrin-A3	Effects of VEGF on capillary-like formation and endothelial cell chemotaxis	[45]
		VEGF		
	miR-486	TLR4	Inhibit TLR4/NF-κB axis, oxidative stress, inflammation, and apoptosis; promote cell proliferation and angiogenesis	[46,47]
	miR-218	HMGB1	Promote endothelial cell proliferation, migration, angiogenesis; reduce inflammatory damage	[48]
	miR-21	PTEN	Inhibit PTEN/PI3K/AKT and apoptosis of endothelial cells, promote angiogenesis	[49,50]
	miR-375	KLF5	Endothelial cell migration, proliferation, germination, and vascular network formation; promote angiogenesis and arteriography	[51]
	miR-214	ATM	Stimulate angiogenesis by silencing neighboring mutant cells with cerebellar ataxia and vascular expansion through microvascular dilation	[52]
		PI3K	Improve viability and tube formation of human endothelial cells damaged by high glucose levels	[53]
		COX-2	Downregulate COX-2/PGE2 axis and target COX-2 antagonized indoxyl sulfate (IS)-induced endothelial cell apoptosis	[54,55]
Anti-angiogenic	miR-34	SirT1	Inhibit Sirt1 to induce aging and impair EPC-mediated angiogenesis	[56]
		Notch1	Inhibit angiogenesis induced by VEGF	[57,58]
	miR-15	CCNB1	Inhibit angiogenesis	[59]

## Data Availability

The data that support the findings of this study are available on request from the corresponding authors [Q.Y. or L.H.] upon reasonable request.

## Abbreviations

The following abbreviations are used in this manuscript:

Exo	Exosome
EC	Endothelial cell
IS	Ischemic stroke
MBVP	Mouse brain microvascular pericyte
MCAO/R	Middle cerebral artery occlusion/reperfusion
tPA	Tissue plasminogen activator
OGD/R	Oxygen–glucose deprivation/reoxygenation
mNSS	Modified neurological severity score
WB	Western blotting
MWCO	Molecular weight cut-off
TEM	Transmission electron microscopy
DLS	Dynamic light-scattering particle
DMEM	Dulbecco’s Modified Eagle’s Medium
CCA	Carotid artery
ICA	Internal carotid artery
ECA	External carotid artery
MCA	Middle cerebral artery
H&E	Hematoxylin–eosin staining
MMPs	Matrix metalloproteinases
